# Cytoskeletal Dynamics: Concepts in Measles Virus Replication and Immunomodulation

**DOI:** 10.3390/v3020102

**Published:** 2011-01-26

**Authors:** Elita Avota, Evelyn Gassert, Sibylle Schneider-Schaulies

**Affiliations:** Institute for Virology and Immunology, University of Wuerzburg, Versbacher Str. 7, 97078 Wuerzburg, Germany; E-Mails: evelyn.gassert@gmx.de (E.G.); s-s-s@vim.uni-wuerzburg.de (S.S.S.)

**Keywords:** measles virus, cytoskeleton, sphingomyelinase

## Abstract

In common with most viruses, measles virus (MV) relies on the integrity of the cytoskeleton of its host cells both with regard to efficient replication in these cells, but also retention of their motility which favors viral dissemination. It is, however, the surface interaction of the viral glycoprotein (gp) complex with receptors present on lymphocytes and dendritic cells (DCs), that signals effective initiation of host cell cytoskeletal dynamics. For DCs, these may act to regulate processes as diverse as viral uptake and sorting, but also the ability of these cells to successfully establish and maintain functional immune synapses (IS) with T cells. In T cells, MV signaling causes actin cytoskeletal paralysis associated with a loss of polarization, adhesion and motility, which has been linked to activation of sphingomyelinases and subsequent accumulation of membrane ceramides. MV modulation of both DC and T cell cytoskeletal dynamics may be important for the understanding of MV immunosuppression at the cellular level.

## 1. Introduction

Measles virus (MV) is the type species of the genus morbillivirus, which are members of the paramyxoviridae. Its replication is confined to humans and—mainly experimentally—to non-human primates, while it is prevented in murine cells due to type I interferons and their effector proteins and other unknown determinants [1]. In humans, the highly infectious MV causes a well known disease in unprotected hosts, usually contracted early in childhood, which is self-limiting and leaves the individual with a life-long protective immunity. Concurrent with efficient virus-specific immune activation, MV causes a generalized, transient immunosuppression allowing for secondary infections to occur and to follow a severe or even lethal course; thereby, constituting the major cause of measles associated infant mortality. Hallmarks of MV-induced immunosuppression are leukopenia affecting most, if not all, peripheral blood mononuclear cell compartments and the inability of lymphocytes to expand in response to activation *ex vivo* [2]. Because the frequency of MV-infected peripheral blood cells is very low (not exceeding 2% at any stage of the disease) [3,4], bystander effects of infected cells on uninfected cells are thought to be major components of lymphocyte inhibition. The model facets, which are investigated from different angles in many laboratories, predict that MV abrogates T cell activation at the level of antigen presenting cell (APC)/T cell communication, also including the ability of these cells to establish functional immune synapses (IS).

## 2. The Role of the Cytoskeleton in MV Uptake and Replication

For host entry, MV crosses the respiratory epithelium, and for this, infects tissue resident professional APCs (e.g., dendritic cells (DCs) or macrophages) rather than epithelial cells. Subsequently, MV accesses secondary lymphatic tissues via infected APCs, where efficient replication along with transmission to lymphocytes occurs, essentially enabling viral dissemination to the reticulo-endothelial system and lymphatic tissues [3,5–7]. Tissue distribution in the hematopoietic system and pathogenesis of wild-type MV infection *in vivo* segregates well with the expression pattern of its entry receptor CD150 (also referred to as signaling lymphocyte activation molecule, SLAM), a member of the Ig family, which is confined to cells of the hematopoietic lineage [8,9]. Attenuated or laboratory adapted MV strains can additionally use CD46, a member of the complement regulator family, to which they bind with higher affinity *in vitro* [10,11]. To what extent interaction with this molecule, which is ubiquitously expressed on all nucleated human cells, determines tissue distribution *in vivo* has not yet been established. CD150 and CD46 also recruit signaling complexes, which regulate both activation of T cells and the polarity of the T cell response [8,12].

**Figure 1.**
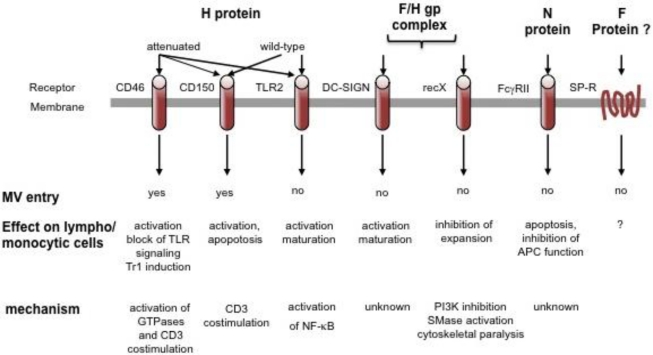
Cellular surface molecules interacting with measles virus (MV). Surface receptors may interact with different MV protein or protein complexes, yet only CD46 and CD150 support MV entry. This may be enhanced by substance P receptor interaction, and, on dendritic cells (DCs), by DC-SIGN which, as TLR2, promotes APC activation on MV binding by relaying signals. Signaling by CD46 and CD150, which can act as CD3 co-stimulators, has mainly been investigated on antibody ligation and has not yet been directly addressed to occur after MV interaction. Signaling via recX and FcγR by MV has been directly linked to inhibition of lympho/monocytic expansion and/or function.

In addition to these characterized entry receptors, wild-type MV interacts and can use other, as yet uncharacterized, receptors to enter endothelial, epithelial and neuronal cells [13–16], while molecules such as substance P receptor or moesin may aid rather than directly promote MV entry [17]. Obviously confined to APCs, DC-SIGN (DC-specific Intercellular adhesion molecule-3-Grabbing Non-integrin) interaction enhances, but does not mediate MV uptake into DCs, and signals in response to MV. This is also true for Toll like receptor 2 (TLR2) on monocytes and may play an important role in monocyte activation along with CD150 upregulation on these cells [18,19]. These interactions all involve the MV hemagglutinin (H) gp alone (for attachment) or in combination with the fusion (F) gp (for entry), which, together as the MV gp complex, also elicit signaling in lymphocytes in a contact dependent manner (see below). In addition, ligation of the Fcγ receptor on B cells and an unknown receptor on lymphocytes by the MV nucleocapsid (N) protein, modulates antibody production, viability or proliferation of target cells (all summarized in Figure 1) [20,21].

**Figure 2.**
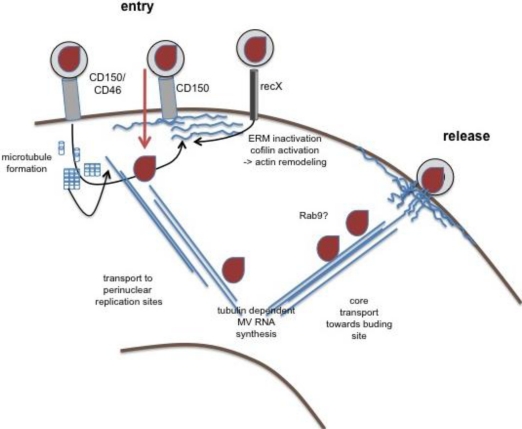
The role of the cytoskeleton in MV replication. Wild-type MV enters into hematopoetic cells after binding to CD150 (or CD46, middle structure). Not yet shown to occur in response to MV interaction, CD46 and CD150 can activate actin remodeling, on ligation. Similarly, recX, when interacting with MV, promotes signals causing dephosphorylation of ezrin-radixin-moesin (ERM) proteins and cofilin, and thereby actin remodeling and microtubule formation (both in blue). Activation of cytoskeletal remodeling may facilitate MV entry and subsequent transport of MV cores (in brown) to perinuclear sites where MV replication occurs, which may rely on tubulin interaction of the replicase. Mechanisms of core transport towards the budding site are unclear, yet there is evidence that it involves vesicular trafficking pathways (sensitivity to Rab9 abrogation). The budding process itself is actin dependent, and barbed ends of actin filaments were detected in association with the nucleocapsid in budding virions by EM.

While at least some of the MV receptor interactions outlined above were clearly linked to modulation of cytoskeletal dynamics (which will be detailed below), little is generally known on the role of the cytoskeleton in MV uptake. The role of cytoskeletal dynamics in MV entry has not yet been analyzed. It is, thus, unknown whether MV attachment and entry involves actin-dependent translocation of trapping molecules along filopodial structures as revealed for HIV, or if clustering of entry receptors (especially CD150, see below) might be required. The impact of actin depolymerizing compounds on viral entry has not been studied as yet. MV gp signaling causes moesin and cofilin dephosphorylation on binding to T lymphocytes [22], which promotes actin remodeling, and this has been suggested to enhance microtubule formation. This has been implicated to facilitate transport of lentiviral cores pseudotyped with MV gps towards the nucleus, and may also be important in trafficking of MV cores [23] (Figure 2). Whether this also refers to the intracellular transport of MV cores (which would then specifically apply to hematopoietic cells) has not been resolved.

Actin is packaged within MV virions, and suggesting a role of actin in virion formation, particle production is cytochalasin B and D sensitive [24,25]. As revealed at the ultrastructural level, MV budding at the plasma membrane shares similarities to generation of microvillar protrusions with regard to actin filament recruitment [26]. These observations corroborated earlier findings where the majority of MV structural proteins were found in association with the actin cytoskeleton in infected cells [27]. Interestingly, actin filaments were found to almost exclusively protrude with their barbed ends into virus particles in close association with the nucleocapsids indicating that MV budding involves vectorial growth of actin filaments. *In vitro*, actin was found associated with purified ribonucleoprotein complexes (RNPs), yet tubulin, rather than actin, co-precipitated with and was required for the activity of the viral polymerase complex, indicating that cytoskeletal components are important at different steps of MV replication [28]. These observations were confirmed in studies in infected Vero cells, which did, however, not reveal targets in the MV replication cycle either [29]. Though likely to occur, microtubular trafficking of MV RNPs involving molecular motors such as dynein or kinesin within infected cells, especially neurons, has not yet been proven. Abrogation of MV release on Rab9 ablation from infected HeLa implies a role of vesicular trafficking pathway for the RNP [30,31] (Figure 2). Overall, the limited amount of data available suggest that, similar to that of related viruses (such as respiratory syncytial virus, RSV) [32], microtubules might play a dominant role in the formation and long distance transport of MV RNPs while actin plays a more prominent role in virus release.

Given the importance of the cytoskeleton network for MV replication and release, it is not surprising that there is no evidence for disruption of the actin-, vimentin- or tubulin network in MV infected tissue culture cells [33]. Spatial alterations of cytoskeletal components such as intermediate and thin (actin) filaments observed in persistently MV infected cells were interpreted as relating to disturbances of RNP transport from assembly sites to the plasma cell membrane, and thereby to persistency.

Because MV infects and relies on motile cells for dissemination, MV infection *per se*, for obvious reasons, does not grossly perturb cytoskeletal dynamics relating to the morphological front-rear polarization (including receptor segregation), acquisition of a motile phenotype and migration. In fact, MV infected DCs and lymphocytes were readily detectable in the majority of lymphoid compartments including the bone marrow in infected rhesus macaques [3,6]. *In vitro*, MV infection of immature DCs promoted phenotypic maturation resembling that driven by TLR4 ligation, and also enhanced Rac-dependent migration of these cells on fibronectin (FN) (mimicking extracellular matrix (ECM) interaction and β1-integrin ligation) [34]. Similarly, MV infection did not grossly affect chemokinesis of lymphocytes through filters, while transendothelial migration of these cells was efficiently abrogated [35], which related to integrin upregulation and activation promoting lymphocyte retention rather than interference with cytoskeletal rearrangement.

## 3. MV Interference with T Cell Signaling and Activation

Cytoskeletal rearrangements essentially govern any step of the formation of an IS where recognition of its cognate peptide/major histocompatibility (MHC) complex on the APC by the T cell receptor (TCR) triggers T cell activation, expansion and finally, differentiation. IS formation is preceded by trapping of T cells on the APC surface, which is followed by ameboid T cell migration for scanning for cognate MHC-peptide complexes, during which they retain their polarized morphology and longitudinal protein gradients, and continuously reorganize their lamellopodia at the leading edge. On antigen recognition, large scale rearrangements of the T cell occur, involving adhesion strengthening by leucocyte function antigen 1 (LFA-1) activation and T cell re-polarization by the segregation of an enlarged, flattened interface with the APC, to which the microtubule organizing center (MTOC) is translocated as well, and, opposite, the distal pole complex [36–38]. Rearrangements of receptors and signaling complexes as well as the formation of localized signaling clusters and their centripetal circulation involves actin cytoskeletal dynamics, which is accompanied by rapid activation/de-activation of ezrin-radixin-moesin (ERM) proteins, which also promote cytoskeletal relaxation as important for conjugate formation [39,40]. In line with the importance of actin dynamics in this process, latrunculin terminates T cell activation as determined by loss of tyrosine-phosphorylation, Ca-mobilization, and conjugate stability, within less than two minutes [41]. In conjugates involving B cells as APCs, the IS acquires a typical ‘bull’s eye’ architecture where central, peripheral and distal supramolecular activation clusters (SMACs) containing CD3/CD28, LFA-1 or CD43, respectively, can be discerned (cSMAC, pSMAC, dSMAC). In contrast, IS involving DCs are predominantly multifocal in that multiple domains concentrating signaling clusters are separated by LFA-1 enriched protrusions, thereby segregating the actual signaling domains [41,42].

Sustained activation of tyrosine kinases, especially Zeta-chain-associated protein kinase 70 (ZAP-70) and lymphocyte-specific protein tyrosine kinase (Lck), and Ras activation are essential for calcium mobilization and Nuclear factor of activated T-cells (NFAT) activation, while sustained activation of the phosphatidyl-inositol-3 kinase (PI3K) pathway provides membrane domains enriched for inositolpolyphosphates (phosphatidyl-inositol-3,4,5-phosphates, PIP_3,4,5_) which serve as docking sites for pleckstrin homology containing proteins such as the Akt kinase and major guanosine exchange factors regulating the activity of small GTPases of the Rho family [41,43]. More recently, semaphorin receptors and their ligands, known to activate GTPases and essentially control adhesion or repulsion by targeting integrin activation and function, have also been implicated in IS function [44]. Within the following sections, we will review how MV might regulate T cell activation independently of infection by interacting with surface receptors.

### Signaling of MV Entry Receptors Independently of Infection

As alluded to above, both CD46 and CD150 are signaling molecules with the property of shaping the quality of lymphocyte responses, which has been extensively studied and reviewed (examples are [8,9,12,45]). Interestingly, both molecules also act as phagocytic receptors with the capacity of routing their cargo into degradative phagosomal compartments and to recruit autophagy-associated components [46,47]. Most likely, MV escapes from degradation because it uses these molecules (CD150 alone for wild-type strains, or both for attenuated strains) not only for binding but also for help in membrane fusion.

A growing number of pathogens, also including human herpesvirus 6, adenovirus and certain Neisseria species, have chosen CD46, a non-raft molecule, as a receptor, and thus, consequences of ligation of this molecule on cellular functions has received much attention in the recent past [12]. CD46 acts to costimulate CD3 in primary human T cells to enhance activation of extracellular signal regulated kinase (ERK), but also to promote morphological alterations and actin relocalization towards the leading edge, compatible with front-rear polarization and acquisition of a motile phenotype [48]. Consistent with lamellopodia formation in these cells, CD46 ligation alone, and more pronounced on CD3 co-ligation, caused tyrosine phosphorylation of Vav and activation of the small GTPase Rac, yet not RhoA or Cdc42 (Figure 2). Obviously, CD46 cytoplasmic domain isoforms generated due to alternative splicing are differentially active in promoting Vav activation on ligation and this affected the degree of morphological alterations and substrate or homotypic adhesion [49]. Since CD46 dependent regulation of cytoskeletal activity in these systems by antibodies were dependent on crosslinking, it is likely that they would also occur on ligation by MV, which would, however, only refer to CD46 use and thereby attenuated strains. The same applies to a potential regulation of ERM protein family members by interaction with CD46. There, physical and functional association of moesin and CD46 in MV uptake were reported, yet the contribution of moesin in this process remained unclear and its activation status and association with the plasma cell membrane and actin cytoskeleton were not analyzed [50]. Though ERM proteins doubtlessly play an important role in cytoskeletal dynamics and receptor segregation in T cell activation [40,51], the importance of CD46 in regulating these molecules in this process in general or its role in modulation of T cell activation by wild-type MV has not yet been substantiated.

As a member and type species of the SLAM family, CD150 (SLAMF1), apart from its role as receptor for MV and microbial sensor for certain gram-negative bacteria (see above), is a self ligand with a strong tendency for lateral self aggregation [8]. Specific adaptors (SLAM-associated protein (SAP) in T, NKT and some B cells, and Ewing Sarcoma Breakpoint region 1/Friend Leukemia Virus Integration-activated transcript 2 (EAT-2) in NK cells, DCs and macrophages) couple CD150 to downstream signaling pathways, which also include recruitment of FynT but also p21 activated kinase (PAK) interacting exchange factor (PIX) [52]. This suggests that CD150 signaling could indeed, via PAK activation, regulate cytoskeletal dynamics (Figure 2). This has, however, so far not been directly revealed, nor has any impact of CD150 ligation on T cell morphing been noted. Not yet verified to occur in T cells as well, CD150 can, on coupling to the 5′lipid phosphatase SHIP-1 (SH2-containing inositol phosphatase-1), activate ERK, and, independently of tyrosine phosphorylation and SHIP-1 recruitment, Akt phosphorylation in B cells [53]. On ligation by antibodies, CD150 potentiated or even induced—in the absence of concomitant TCR engagement—cytokine release from human T cells or promoted cytotoxicity of CD8+ T cells collectively, rendering it an overall efficient T cell co-stimulator. Consequences of MV ligation for CD150 signaling are as yet unknown.

## 4. MV Surface Interaction with T Lymphocytes: The Cytoskeletal Collapse and Role of Sphingomyelinase Activation

Almost 15 years ago it became clear that surface interaction with the MV gp complex abrogated stimulated or—in cell lines—constitutive proliferation of hematopoietic cells [54]. This did not involve CD150 or CD46, membrane fusion, infection, apoptosis or release of soluble inhibitory factors. Rather, cells exposed to the MV gp complex on infected cells, cells transfected to express MV F/H proteins or inactivated virions, arrested in the G_1_ phase of the cell cycle associated with typical deregulation of cyclin dependent kinases and their inhibitors, provided the F protein was proteolytically activated [55–59]. Importantly, the MV gp complex also caused similar effects in experimentally infected cotton rats [60,61].

**Figure 3.**
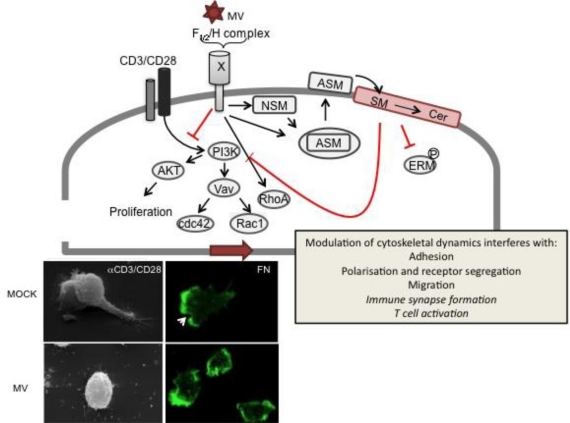
MV signaling via receptorX causes T cell actin cytoskeletal paralysis. On binding to recX, the MV gp complex (consisting of the proteolytically activated F_1/2_ heterodimer and the H protein) signals to abrogate PI3K activation, e.g., in response to TCR ligation (CD3/CD28), which causes inactivation of downstream effectors such as Akt kinase, Vav and Rac1/Cdc42. To a major extent, PI3K interference is mediated by sequential activation of neutral (NSM) and acid sphingomyelinase (ASM), which promotes surface translocation of ASM and subsequent breakdown of outer membrane sphingomyelin (SM) and formation of ceramide (Cer)-enriched membrane microdomains. ASM activation and ceramide accumulation have been linked to abrogation of ERM phosphorylation, RhoA activation and PI3K interference and loss of actin based protrusions in non-lymphoid cells. In T cells, ceramide induction by MV or recombinant bacterial sphingomyelinase has been directly linked to the MV (bottom panels) loss of actin cytoskeletal dynamics as evidenced by loss of actin and receptor polarization, microvillar protrusions or lamellopodial structures on substrates mimicking the ECM (such as fibronectin, bottom right panels, where actin is detected by phalloidin (green), the arrow marks the leading edge in MOCK treated cells) or the immunological synapse (αCD3/CD28 coated slides, bottom left panels; the upper panel shows two interacting MOCK treated T cells). Whether ceramide accumulation also directly accounts for MV-dependent inhibition of IS formation or T cell activation is as yet unknown (therefore indicated in italics).

On a molecular level, MV gp signaling was found to target activation of the PI3/Akt kinase pathway in response to IL-2R or TCR triggering [62] (Figure 3). Later, this was related to both delayed degradation of Cbl-b, or, generated by alternative splicing due to PI3K inhibition, a constitutively active SHIP-1 isoform, which continuously depleted the plasma membrane PIP_3,4,5_ pool thereby raising the threshold for activation [63,64]. Vav is a downstream effector of PI3K and, thus, MV signaling targeted TCR-dependent activation of Rac1 and Cdc42, yet not RhoA which was even slightly activated on MV exposure. Indicating that MV contact abrogated actin cytoskeleton reorganization, the ability of T cells to adhere to and polarize on FN or α-CD3/CD28 coated substrates was strongly impaired as was their ability to translocate the MTOC towards the IS and CD3 to the cSMAC in conjugates with lipopolysaccharide(LPS)-matured DCs [22].

More recently, we were able to show that TCR-mediated IS recruitment of plexinA1 and neuropilin-1, a semaphorin receptor complex, which is involved in early IS formation and function, is also ablated in MV exposed T cells [65–67]. As evidenced in neuronal cells, translocation of these molecules and the activity of the signaling moiety, plexinA1, to regulate adhesion, involve cytoskeletal dynamics and integrin signaling, respectively [68]. PlexinA1/neuropilin-1 are expressed both on T cells and DCs, and thus, their effect to promote early IS functions may involve homotypic interaction, though heterotypic have not been excluded. Late after DC activation, soluble semaphorin 3A (SEMA3A) is produced, which acts as a repulsive ligand promoting collapse of actin based structures, e.g., in neuronal growth cones, and this involves adaptor proteins also referred to as ‘collapsins’ [69,70]. In line with its function in resolving cell contacts, SEMA3A production late after DC/T cell interaction has been related to termination of activation [71,72], and it is thus remarkable that in co-cultures of MV-infected DCs and T cells, this repulsive semaphorin receptor ligand is produced already within the first hours. Recombinant SEMA3A (and SEMA6A) caused a marked transient loss of cellular actin and actin based protrusions, yet only moderately affected DC/T cell conjugate stability and efficiency *in vitro* [67,72], and thus, the role in regulating IS functions and potential modulations thereof by viruses remains to be established. Semaphorin production may, however, not essentially contribute to the instability of conjugates formed between MV-infected DCs and T cells. The majority of conjugates observed in these co-cultures resolved within less than two minutes and only allowed for a transient Ca-burst instead of sustaining both the cell contact and Ca-mobilization as required for T cell activation. Indicating that again the MV gp complexes essentially accounted for loss of IS stability, this was largely rescued when DCs infected with a recombinant MV expressing VSV G protein instead were used in conjugate formation [34].

MV exposure does not only affect stimulated cytoskeletal rearrangements, yet causes a loss of actin based microvillar structures from T cells, and this was accompanied by a loss of ERM protein phosphorylation [22] (Figure 3). These observations, together with RhoA activation and PI3K interference (see above) were consistent with those made mainly in non-T cells after activation of spingomyelinases (SMases). These differ in their respective pH optimum, and their activity largely accounts for accumulation of membrane ceramides; that of the acid sphingomyelinase (ASM) catalyzes the transformation of small membrane cholesterol and sphingomyelin (SM) enriched microdomains (also referred to as rafts) into large, ceramide-enriched membrane platforms in response to a variety of external stimuli [73,74]. In hematopoietic cells, these include ligation of death receptors CD40, FcγRII and CD28 [75,76]. Pathogens such as *P. aeruginosa*, *S. aureus* and rhinovirus promote and rely on formation of ceramide-enriched platforms for entry (reviewed in [75]) while fusion-dependent uptake of HIV is prevented on ceramide accumulation [77,78]. Ceramide enriched platforms regulate lateral diffusion and recruitment of surface receptors and membrane-proximal signaling complexes, thereby enhancing initiation or modulating signaling pathways including PI3K [79,80]. SMase activation was also implicated in cytoskeletal remodeling in breast cancer MCF-7 cells [81,82]. There, cisplatin induced ASM activation and subsequent sphingomyelin breakdown in the outer membrane bilayer promoted a protein phosphatase 2A (PP2A)-dependent loss of ERM protein phosphorylation and of actin based protrusions.

Indeed, MV exposure was found to sequentially activate NSM and ASM in T cells, and this accounted for the general loss of actin cytoskeletal dynamics in these cells [83] (Figure 3). Thus, pharmacological or genetic ablation of SMase activation essentially restored the ability of MV exposed T cells to polarize and spread on substrates, segregate receptors, migrate in response to stromal cell derived factor 1 (SDF-1) and to retain actin-based microvillar protrusions. In turn, exogenous ceramide accumulation caused loss of polarity and ezrin, yet not moesin phosphorylation on stimulation indicating that SMAse activation in general acts to regulate T cell activation by modulating cytoskeletal dynamics.

## 5. Outlook: SMase Activation in Immune Cells—Is There More to It?

As outlined above, SMase activation in response to receptor triggering may promote receptor clustering favoring subsequent internalization [73,75]. Because lateral concentration of viral receptors has been proposed to be one of the principles underlying enhancement of viral DC infection by DC-SIGN [84], we addressed the possibility that SMase activation could play a role in this process. Indeed, ligation of DC-SIGN by antibodies, mannan or MV caused activation of both NSM and ASM within few minutes and subsequent outer membrane display of ceramides [85]. SMase activation was found to be essential for membrane proximal relay of DC-SIGN signaling as reflected by abrogation of Raf-1 and ERK-1/2 phosphorylation known to occur in response to DC-SIGN ligation [86–88] by SMase inhibitors, but also for promoting enhancement of MV uptake into DCs. This was linked to SMase-dependent translocation of CD150 from intracellular storage compartments for transient membrane display [85]. Though not analyzed to occur in DCs, HIV fusion mediated uptake was found to be inhibited rather than enhanced by accumulation of membrane ceramides because this interfered with lateral CD4/chemokine receptor co-segregation [77,78,89]. It is tempting to speculate that—in case HIV would also cause DC-SIGN dependent SMase activation there—lateral segregation of its entry receptors on DCs might favor enhancement of internalization and storage of viral particles for subsequent trans-infection of T cells. Thus, SMase activation by attachment receptor interaction might be highly relevant for the mode of viral uptake into DCs and subsequent delivery to T cells and thereby viral pathogenesis.
